# Ruminal and Fecal Bacteriome of Dairy Calves Fed Different Levels and Sources of NDF

**DOI:** 10.3390/ani11092705

**Published:** 2021-09-16

**Authors:** Gercino Ferreira Virgínio Júnior, Ana Paula da Silva, Ariany Faria de Toledo, Milaine Poczynek, Amanda Moelemberg Cezar, Horácio Montenegro, Luiz Lehmann Coutinho, Carla Maris Machado Bittar

**Affiliations:** Department of Animal Science, Luiz de Queiroz College of Agriculture, University of São Paulo, Av. Pádua Dias, 11. Piracicaba, São Paulo 13.418-900, Brazil; annaps@usp.br (A.P.d.S.); arianytoledo@usp.br (A.F.d.T.); milainepoc@gmail.com (M.P.); amandamcezar@usp.br (A.M.C.); h.montenegro@gmail.com (H.M.); llcoutinho@usp.br (L.L.C.)

**Keywords:** calf nutrition, fecal microbiota, hay, microbiome

## Abstract

**Simple Summary:**

The present study aimed to characterize and understand how the gastrointestinal microbiome (in rumen and feces) of pre-weaning dairy calves is affected by feeding a starter concentrate with two levels of fiber and the inclusion of hay in the diet. Another study objective was to verify if the effects on the microbiome remained after weaning. The ruminal bacteriome was not affected by levels or sources of fiber. The fecal bacteriome was affected by age and diet. The inclusion of hay affected the fecal microbial diversity.

**Abstract:**

A starter concentrate containing different levels and sources of NDF can modify the gastrointestinal bacteriome. This study evaluated 18 Holstein calves housed in un-bedded suspended individual cages, fed one of three treatments: 22NDF: a conventional starter containing 22% NDF (n = 7); 31NDF: a starter with 31% NDF, replacing part of the corn by soybean hull (n = 6); and 22Hay: diet 22NDF plus coast-cross hay ad libitum (n = 5). All animals received 4 L of milk replacer daily, weaned at 8th week of age, and housed in wood shelters until week 10. To evaluate the bacteriome, the bacterial community of ruminal fluid and fecal samples was determined by sequencing V3 and V4 region amplicons of the 16S rRNA gene. Bacterial diversity in rumen was not affected by diet or age. The phyla Firmicutes and Bacteroidota, and *Prevotella*’ genus were the most abundant in ruminal fluid and fecal samples. In feces, the α-diversity indices were higher for 22Hay. All indices were significantly affected by age. We believe that the ruminal bacteriome was affected by basal diet components, but not affected by NDF levels or sources. The supply of hay was effective in modifying the fecal bacteriome of dairy calves due to hind gut fermentation.

## 1. Introduction

The rumen has been the focus of further research with adult ruminants. Likewise, many studies with young calves have focused on the development of ruminal function and microbial succession [1,2,3,4]. Therefore, in recent years, the gut microbiota has also been the subject of studies by analyzing fecal samples [5,6,7]. However, how the microbiota colonizes and establishes itself in the gastrointestinal tract (GIT) in the pre-weaning calves has not been fully elucidated [8].

Knowing and understanding how microorganisms are established in the GIT has important ecological and physiological implications on calf development and health and, also, on the long-term animal productive efficiency [1]. Additionally, understanding how the feeding and management can affect the ruminal and gut microbiota can be a window of opportunity for improving animal performance and health.

The diet, especially the solid diet, represents one of the most important factors affecting the bacteriome [9]. Thus, the composition of the starter concentrate has a great influence on bacterial composition and ruminal development [10,11,12], as it serves as a constant inoculum for microorganisms and also as a substrate for their growth. Starter concentrates containing a greater fiber proportion can promote a higher cellulolytic bacterial growth, ruminal protozoa and, consequently, a higher acetate:propionate ratio, whereas a higher starch diet causes contrasting effects, such as increased amylolytic bacteria, as well as a higher butyrate and lactate concentration in the ruminal environment [11].

The composition of ingredients and nutrients in starter concentrates varies widely. The starch-rich concentrate is rapidly fermentable by the microbiota, causing a decrease in ruminal pH and, thus, it can inhibit the microbial growth of more sensitive groups at a lower pH. Thus, a minimum fiber content is essential to maintain a ruminal environment suitable for microorganisms and produce healthy calves that can respond to different feeding systems [13]. Davis and Drackley [14] suggest starter concentrates containing 16 to 25% NDF. This wide range of recommendations aims to ensure a highly digestible material, and also ensure the health of the ruminal epithelium. However, the ideal fiber content in the diet of pre-weaning calves is still a matter of debate [15]. Moreover, it is unclear how the GIT microbiome responds to these variations of NDF in the starter concentrate.

The inclusion of hay in the pre-weaning calves’ diet could be an alternative, since it can stimulate the establishment of specific bacterial populations, such as cellulolytic bacteria [16,17,18]. Another alternative is the inclusion of ingredients with high digestibility fiber replacing corn, such as soybean hulls [19] or citrus pulp [20], and can be used as an energy source, while maintaining the adequate fiber diet content and reducing the lactate concentration [21]. However, it is unclear how these ingredients could affect the GIT microbiota of pre-weaning calves.

This study aims to analyze the gastrointestinal bacteriome of dairy calves fed a starter concentrate with different levels (22, 31, or 22% plus the supply of hay) and sources (soybean hulls or hay) of NDF in the pre and post-weaning phases. We hypothesized that the solid diet composition may have significant effects on the ruminal and fecal bacterial community, either by increasing the proportion of soybean hulls in partial replacement to corn in the starter concentrate containing a higher level of NDF (31%), or by providing hay with an intermediate NDF starter concentrate (22%).

## 2. Materials and Methods

### 2.1. Animals, Calf Facilities and Feeding

This study was conducted at the calf facilities of the Department of Animal Science, “Luiz de Queiroz” College of Agriculture, São Paulo University, located in Piracicaba—São Paulo, Brazil.

This bacterial community study was part of another study with 35 newborn Holstein calves (22 males and 13 females; birth weight 37.33 ± 1.33 kg) evaluated in a randomized block design during pre-weaning, and performance data, health, and ruminal and blood parameters have already been published by Poczynek et al. [13]. From these 35 calves, 18 calves (36.31 ± 1.35 kg) were randomly selected and used for evaluation of bacterial community.

All calves were fed 4 L of high-quality colostrum (>50 g/L) in the first 6 h of life [22], and all animals had serum protein above 5.5 g/dL at 48 h of life [23]. The experimental period was 70 d.

From birth to 56 d, the animals were housed in individual suspended cages (113 cm × 140 cm), in an airy shed, using a rubber mat as a bed. The animals had free access to water and starter concentrate in individual troughs located at the front of the cage.

The milk replacer (23.8% CP and 28.5% EE, Sprayfo Azul, Sloten de Brazil Ltd., Santos, SP, Brazil) was diluted to 12.5% solids, supplied in buckets, in the volume of 4 L per day, divided into two meals (7 h and 17 h). As described by Poczynek et al. [13], this low volume of liquid diet was to stimulate the earlier starter intake so that the effects of the solid diet were more pronounced.

Three solid diets were evaluated, based on two initial concentrates (Table 1), formulated according to the NRC [24] to meet the demands of pre-weaning calves: (1) 22NDF: starter concentrate containing 22% NDF (control; n = 7); (2) 22Hay: starter concentrate containing 22% NDF, with the inclusion of additional fiber source, such as coast-cross hay chopped ad libitum in a separate bucket from the concentrate (n = 5); (3) 31NDF: starter concentrate containing 31% NDF, with increasing proportion of soybean hulls in partial replacement to corn (n = 6). Thus, a comparison was established among NDF levels, 22% vs. 22% + hay vs. 31%, and a comparison between fiber sources, soybean hulls vs. coast cross hay. As described by Poczynek et al. [13], average solid diet intake during the pre-weaning period was 273.7 (22NDF), 385.6 (31NDF), and 293.4 g/d (Starter, 293.422 + Hay 15.5; 22Hay). At weaning, feed intake for each diet was 617.0 (22NDF), 876.4 (31NDF), and 697.6 g/d (Starter, 647.0 + Hay, 50.51; 22Hay).

Weaning began at 56 d of age on the afternoon feeding, reducing the supply of milk replacer by 0.5 L per meal, until complete weaning at 58 days. After weaning, the animals were housed in wood shelters until 70 d of age, with free access to water. The same starter concentrate continued being offered in post-weaning phase and chopped hay was offered for all groups. The offer of hay after weaning for all groups represents a modification in experimental conditions of the diet compared to the pre-weaning period. After weaning, all diets had an additional fiber source; however, a difference in fiber levels in the solid diet continued to be observed. Intake data for this period were not available.

### 2.2. Evaluation of Bacterial Community

#### 2.2.1. Ruminal Fluid and Fecal Samples Collections

Samples of ruminal fluid were collected at 14 (2nd week), 28 (4th week), 42 (6th week), 56 (8th week; weaning), and 70 (10th week; post-weaning) days of life, two hours after morning feeding. The samples were collected via oro-esophageal, using a flexible hose 150 cm in length, 1.3 cm in internal diameter, and 0.2 cm in wall thickness, which was connected to a vacuum pump (Model TE-0581, Tecnal Ltd.a., Piracicaba, SP, Brazil). The samples were collected, discarding the initial portion to avoid saliva contamination. After filtering on cotton fabric, an aliquot was stored in a sterile 2 mL plastic tube, and frozen in a freezer at −20 °C for further evaluation of the microbiome.

Fecal samples were collected on day 0 (±2 h after birth, before colostrum feeding) and at 7 (1st week), 14 (2nd week), 28 (4th week), 56 (8th week) and 70 (10th week) days of life. The samples were collected manually, aseptically, directly from rectum of the animals, with latex glove, changing gloves at each collection to avoiding cross-contamination among samples. Approximately 2 g of feces were collected in sterile tubes, and immediately frozen at −20 °C.

In case of a lost sample on the collection day of ruminal fluid or feces, due to delay in the collection or absence of material (fecal), a new try was conducted on the following day, and if the sample was lost again, this collection was discarded and not repeated.

#### 2.2.2. DNA Extraction, Library Preparation, and Sequencing

DNA extraction from the ruminal and fecal samples was conducted using the QIAamp^®^ Fast DNA Stool Mini Kit extraction kit (QIAGEN, Hilden, Germany), following the modifications suggested by Yu and Morrison [25]. The DNA sample quality of the bacterial community was evaluated by 0.8% agarose gel electrophoresis and the concentrations were quantified in a NanoDrop^®^ ND-2000 spectrophotometer (Thermo Fisher Scientific, Wilmington, DE, USA).

The libraries were prepared following the Illumina recommendations. Bacterial locus-specific primers were used for amplification flank of the V3–V4 region. Overhang sequence of adapters was included in locus-specific primers. Illumina adapter sequences were:16S Amplicon PCR Forward Primer: 5’ TCGTCGGCAGCGTCAGATGTGTATAAGAGACAGCCTACGGGNGGCWGCAG;16S Amplicon PCR Reverse Primer: 5’ GTCTCGTGGGCTCGGAGATGTGTATAAGAGACAGGACTACHVGGGTATCTAATCC.

Sequencing was performed on Illumina MiSeq system (Illumina, San Diego, CA, USA) and produced readings were 2 × 250 bp.

#### 2.2.3. Bioinformatic Analyses

The data were analyzed as a previously published pipeline [26], using a set of packages implemented in the R language (https://www.R-project.org/) and available through the Bioconductor project [27,28].

The multiplexed readings were first assigned to biological samples. The DADA2 program [29] was used for modeling and amplicon errors correcting, without building OTUs. The FASTQ files were filtered to cut the primers’ PCR sequences and filter out the 3 ‘ends of reads due to quality decay (Q < 30), but maintaining the overlap for subsequent joining of the reads and reassembly of the V3–V4 region fragments. Following DADA2’s initial processing of the sequencing data, to each ASV (Amplicon Sequencing Variants), taxonomies were assigned using a DADA2 program implemented of the naive Bayesian classifier method used for this purpose [30]. The SILVA database was used as a reference [31].

The taxonomical classifications produced by DADA2, as well as its quantifications, were imported to the phyloseq program [32], also implemented in R. Alpha and Beta diversity analyses were performed in the phyloseq package as described by Callahan et al. [29]. A permutational multivariate analysis of variance (PERMANOVA) was performed for the Beta diversity analysis, using weighted UniFrac distances, testing for the effect of treatment, week, and its interaction. ASVs not classified to at least the family level were filtered out and ASVs marked as the same species were clustered. Having applied these filters, raw abundance tables and relative abundance counts were obtained.

The taxonomic counts within the phyloseq object were imported to the edgeR package [33] to normalize the library sizes of each sample [34] and, subsequently, the counts were transformed to the 2-base logarithm of the counts per million (logCPM) of each sample (voom transformation) [35]. These transformations allowed the linear models, as implemented in the limma package [36], to be used in the differential abundance analysis. After the linear model was fitted with limma, the differential taxonomic abundance was tested for each contrast (pair of treatments) with moderate *t*-tests [37].

## 3. Results

Sequencing information (number of raw reads, number of quality-filtered reads, number ASVs identified) is shown in Supplementary Tables S1–S3.

### 3.1. The Bacterial Community in Ruminal Fluid

The diversity indices, Shannon, Simpson, Chao1 (richness) and Pielou (evenness) of the bacterial community in the rumen were not affected by the different solid diets or age of the animals, as well as by the interaction between these factors (Table 2).

**Figure 1:**
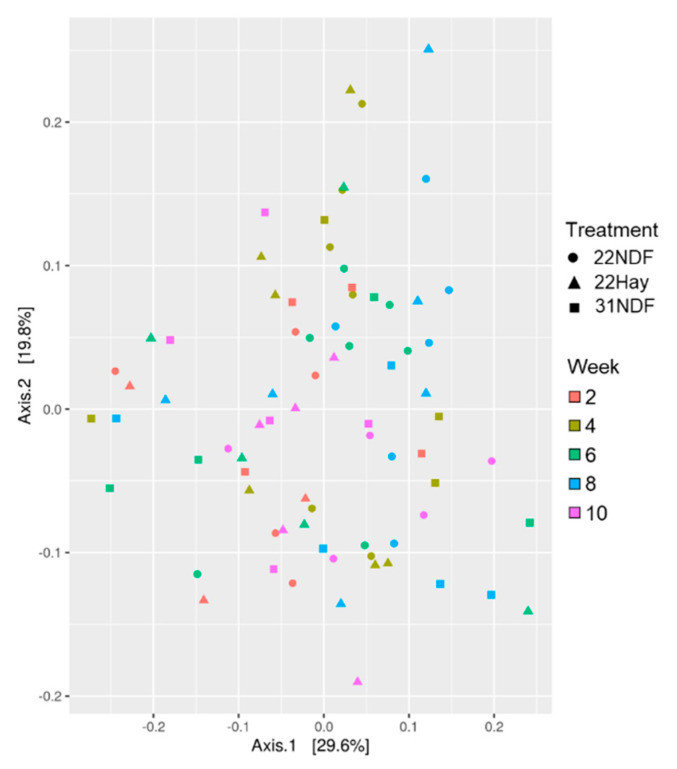
β-diversity of bacterial community in ruminal fluid samples in dairy calves fed different levels and sources of NDF. Multidimensional scaling (MDS) showing the weighted UniFrac distance metric; 22NDF—calves fed starter concentrate with 220 g NDF/kg; 22Hay—calves fed starter concentrate with 220 g NDF/kg and grass hay; 31NDF—calves fed starter concentrate containing 310 g NDF/kg.

The β-diversity analysis also did not show the effects for solid diets (*p* = 0.778), age (*p* = 0.117), or the interaction between these factors (*p* = 0.819) (Figure 1).

**Figure 2:**
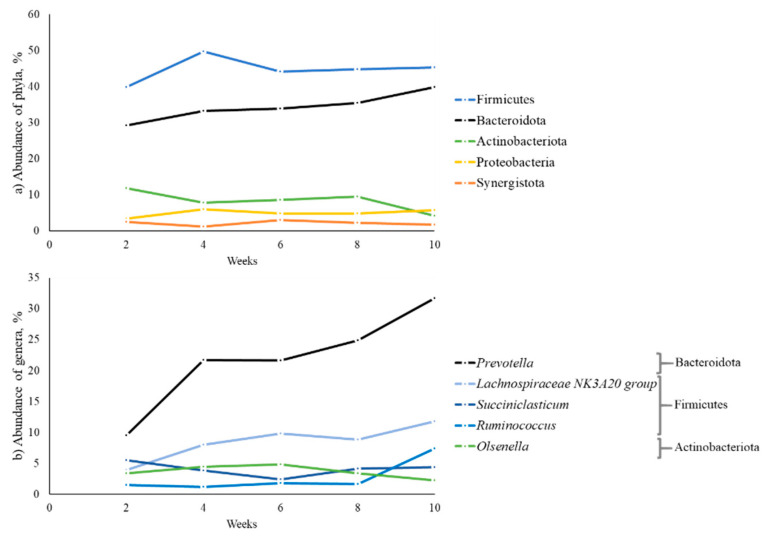
Relative abundance of bacterial phyla and genera in ruminal fluid samples during pre- and post-weaning in dairy calves fed different levels and sources of NDF.

Sixteen bacterial phyla were identified in ruminal fluid samples, and the most abundant phyla were Firmicutes (44.74%), Bacteroidota (34.33%), Actinobacteriota (8.39%), Proteobacteria (4.98%), and Synergistota (2.16%). All abundance averages, per treatment and per week, are in the Supplementary Table S4. The abundance of these phyla during the 10 weeks can be seen in Figure 2a. Firmicutes was the most abundant throughout the period. Actinobacteriota decreased and proteobacteria increased in abundance at week 10.

Of these 16 phyla, 259 genera were quantified. Among these genera, the most prevalent were *Prevotella* (21.87%), the *Lachnospiraceae NK3A20 group* (8.47%), *Succiniclasticum* (4.08%), *Olsenella* (3.69%), and *Ruminococcus* (2.72%), corresponding to 40.83% of the total (Supplementary Table S5). The variation in the abundance of these genera can be seen in Figure 2b.

**Figure 3:**
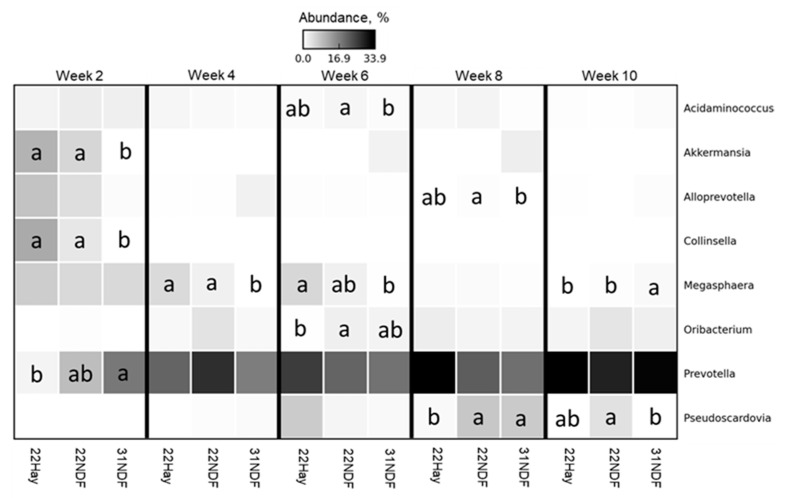
Differential abundance in most abundant bacterial genera in ruminal fluid samples of dairy calves fed different levels and sources of NDF. Data are visualized as heatmap. Comparisons are among treatments within week. Means followed by the same letter are not significantly different by the *t*-test (*p* > 0.05). Genera rows without letters were not significantly different; 22NDF–calves fed starter concentrate with 220 g NDF/kg; 22Hay—calves fed starter concentrate with 220 g NDF/kg and grass hay; 31NDF—calves fed starter concentrate containing 310 g NDF/kg.

All taxonomic abundance differential data were reported in the Supplementary Table S6. Figure 3 shows eight bacterial genera at a relative abundance of ≥1%, as a heat-map. A few bacterial genera had an abundance above 1%. *Acidaminococcus*, *Akkermansia*, *Alloprevotella*, *Collinsella*, and *Prevotella* showed differences in only one of the weeks evaluated.

### 3.2. Fecal Bacterial Community

**Figure 4:**
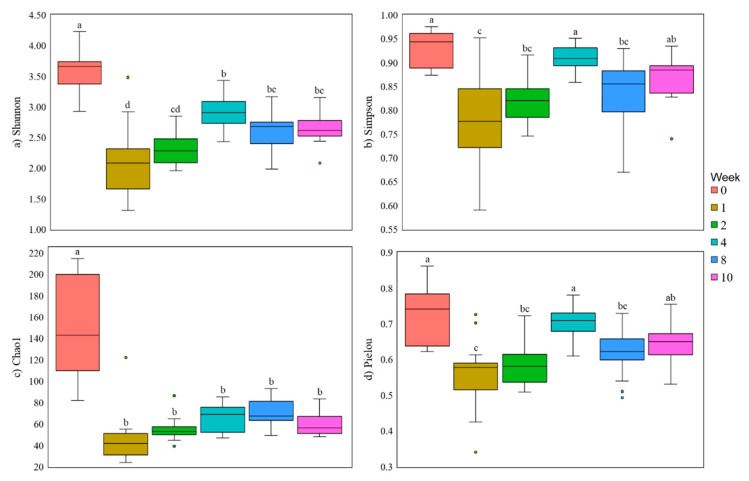
α-diversity indices in fecal samples from dairy calves fed different levels and sources of NDF. Letters above boxes indicate significant differences at *p* > 0.05. Data are visualized as box-plots showing the median and the interquartile (midspread) range (boxes containing 50% of all values), the whiskers (representing the 25 and 75 percentiles), and the extreme data points.

Fecal microbial diversity showed results very different from those found in the ruminal bacterial community. The Shannon and Pielou indices (diversity and evenness, respectively) were higher in samples of animals receiving the 22Hay diet when compared to the 22NDF diet, not differing from the 31NDF diet (*p* = 0.048 and *p* = 0.007). Simpson tended to have the same effect seen in Shannon and Pielou indices (*p* = 0.072). All indices were affected by age (Figure 4, *p* < 0.001); however, there was no effect of the interaction between the solid diet and the age of the animals (Table 3).

In general, the samples collected at time 0 (meconium) had a higher diversity, richness, and evenness. Week 1 had less diversity and evenness. Shannon and Simpson’s diversity and Pielou’s evenness increased until week 4. Richness (Chao1) did not differ among weeks after week 0 (Figure 4).

**Figure 5:**
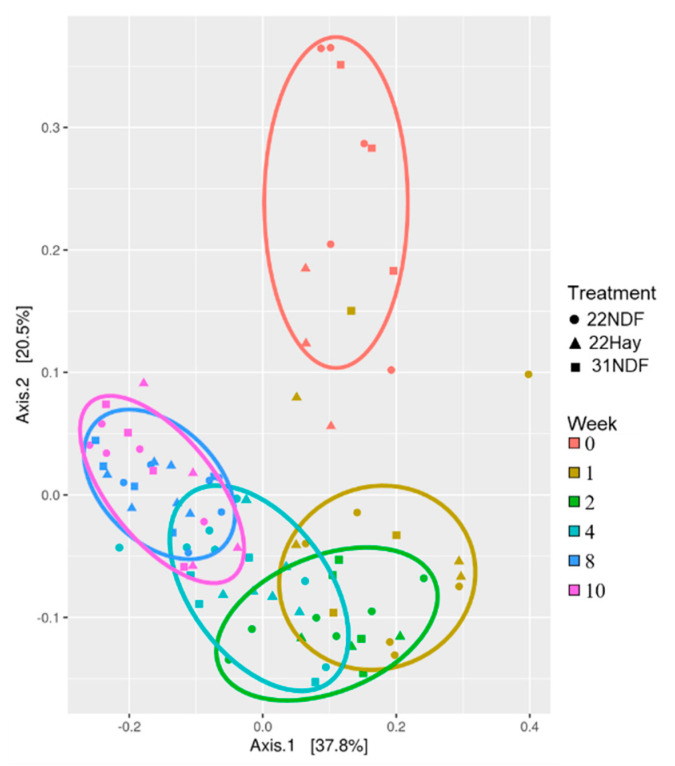
β-diversity of bacterial community in ruminal fluid samples in dairy calves fed different levels and sources of NDF. Multidimensional scaling (MDS) showing the weighted UniFrac distance metric; 22NDF—calves fed starter concentrate with 220 g NDF/kg; 22Hay—calves fed starter concentrate with 220 g NDF/kg and grass hay; 31NDF—calves fed starter concentrate containing 310 g NDF/kg.

The β-diversity showed that there was an effect only for the animals’ ages (Figure 5; *p* < 0.001), with no effect of the diets or the interaction of these factors (*p* = 0.433; *p* = 0.114, respectively).

**Figure 6:**
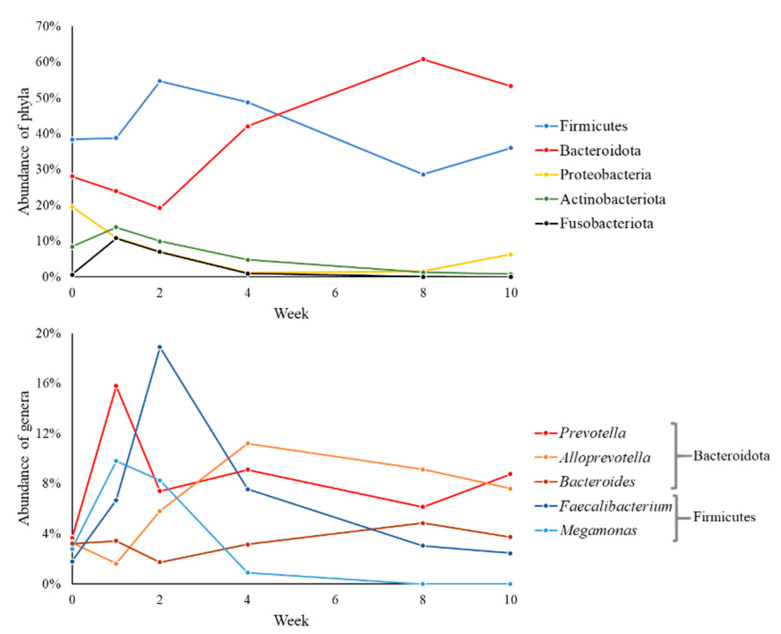
Relative abundance of bacterial phyla and genera in fecal samples during pre- and post-weaning in dairy calves fed different levels and sources of NDF.

Twenty-four bacterial phyla were identified in the fecal samples. Firmicutes (40.92%), Bacteroidota (37.94%), Proteobacteria (7.82%), Actinobacteriota (6.56%), and Fusobacteriota (3.26%) were the most abundant phyla corresponded to 96.50% of the total (Supplementary Table S7). Firmicute was the dominant phylum until 4 weeks, being surpassed in abundance by the phylum Bacteroidota in week 8 (Figure 6). Proteobacteria, Actinobacteriota, and Fusobacteriota remained during the evaluated period with an abundance below 10%. Within the 24 phyla, 555 genera were identified and *Prevotella* (8.49%), *Faecalibacterium* (6.75%), *Alloprevotella* (6.46%), *Megamonas* (3.63%), and *Bacteroides* (3.56%) were the most abundant genera (Supplementary Table S8). *Prevotella* was the most abundant in weeks 1 and 10 (15.80 and 8.78%, respectively), *Faecalibacterium* in week 2 (18.91%), and *Alloprevotella* in weeks 4 and 8 (11.21 and 9.14%, respectively).

**Figure 7:**
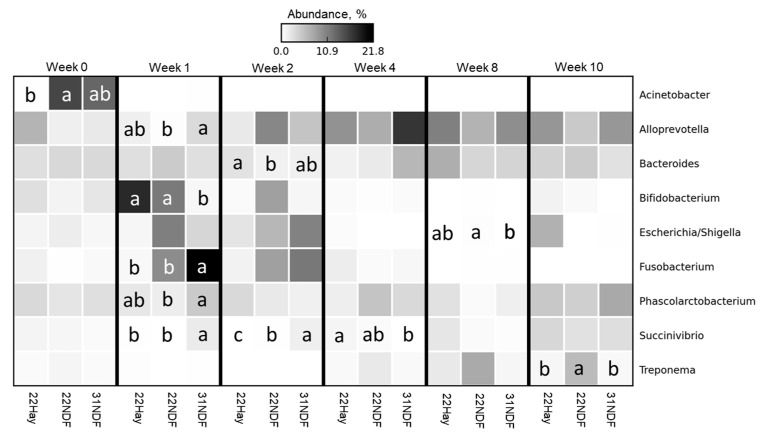
Differential abundance in most abundant bacterial genera in fecal samples of dairy calves fed different levels and sources of NDF. Data are visualized as heatmap. Comparisons are among treatments within week. Means followed by the same letter are not significantly different by the *t*-test (*p* > 0.05). Genera rows without letters were not significantly different; 22NDF—calves fed starter concentrate with 220 g NDF/kg; 22Hay—calves fed starter concentrate with 220 g NDF/kg and grass hay; 31NDF—calves fed starter concentrate containing 310 g NDF/kg.

All taxonomic abundance differential data were reported in the Supplementary Table S9. Figure 7 shows nine bacterial genera at a relative abundance of ≥1%, as a heatmap. A few bacterial genera had an abundance above 1%. Eight genera showed differences in only one of the weeks evaluated. Only *Succinivibrio* showed differences in more than one week, 31NDF had more abundance of this genus in week 1 and 2; however, it had a lower abundance in week 4 when compared to 22Hay. In other weeks, there were no differences among treatments.

## 4. Discussion

### 4.1. Ruminal Microbial Diversity

According to Malmuthuge and Guan [38], the microbial diversity in rumen is very dependent on the diet, and the substrate available to be fermented. Additionally, diversity has a strong specificity with the host. Dias et al. [4] found a greater ruminal microbial diversity for animals fed with a starter concentrate during the pre-weaning phase compared to animals that were fed exclusively with whole milk. Biscarini et al. [39] also found a higher diversity for animals supplemented with grape-pomace compared to the control group, both diets with a similar NDF (20%) level to one of the diets in the present study. However, in the present study, even the different levels and sources of NDF were not sufficient to alter the ruminal bacterial diversity. Animals supplemented with hay were expected to have a higher ruminal diversity, as what occurred in the study by Kim et al. [40]. Even after weaning, when all animals received hay to complement their feed, the bacterial diversity was unaffected. As noted by Poczynek et al. [13], the different diets, in addition to not affecting the starter intake, which was below expectations for animals fed only 4 L per day of a liquid diet, also did not affect most ruminal parameters, except for N-ammoniacal. These results in Poczynek’s study corroborate the data found in our study.

Previous studies have reported that bacterial communities in the rumen change with age [2,41,42]. Jami et al. [2] suggested a transition from a heterogeneous and less distinct community to a more homogeneous and diverse mature bacterial population as adults. In the study by Dill-McFarland et al. [42], calves sampled a few days after weaning had a more diverse ruminal community compared to calves sampled during the pre-weaning phase. This did not occur in the present study since the microbial diversity did not vary throughout the pre-weaning phase and remained similar in the post-weaning period.

A possibility for the absence of effect in the microbial diversity is the presence of monensin in the mineral/vitamin premix. Monensin is an ionophore that interrupts the flow of ions through microbial membranes, causing cell death [43]. In addition to being used to control coccidiosis [44], in ruminants, it is used to limit the effects of ruminal acidosis [45]. However, there are no studies in the literature that assess whether the adequate monensin concentration to treat coccidiosis can affect the establishment and development of the ruminal microbiome in pre-weaning dairy calves.

In beef steers, the association of monensin (478.3 mg/kg of DM) and Tylosin (96 mg/kg of DM) decreased the ruminal microbial diversity when compared to animals that never received any type of ionophore, beta-agonists, antibiotics or antimicrobials [46]. Another important point observed by authors was that the phylum level composition was not affected by the treatments.

In the present study, the concentration of monensin was 36 mg/kg of DM. This concentration was the range of the recommendation cited by Bangoura and Bardsley [44] for the treatment of coccidiosis, from 0.3 to 1.0 mg/kg body weight. Even though the concentration was 10 times lower than that used in the study by Thomas et al. [46], perhaps, calves with a developing rumen, the concentration present in the diet, already caused effects on the bacterial community in establishment.

Until the introduction of modern molecular techniques, it was believed that a functional population of bacteria in the rumen of calves would be established at 10 to 14 days of age when there was access to a conventional starter concentrate [47]. Today, it is clear that some functional microorganisms in the rumen are present, in less abundance, since the first day of life [48,49], even before the introduction of a solid diet [41]. In the present study, the evaluation of the ruminal bacteriome began at 14 d, and all animals already had a functional bacterial community even with a very reduced starter intake, of approximately 50 g, as described by Poczynek et al. [13]. Thus, the rumen already had a microbiota with a composition that remained stable after the animals were weaned.

### 4.2. Fecal Microbial Diversity

Pre-weaning calves are considered to be pre-ruminants since the rumen is not fully developed and fully functioning as a fermentation chamber. Thus, part of the diet ingested is not fermented by the rumen, and can serve as a substrate for microbial growth in the hindgut [42] and, thus, these higher nutrients flow as a function of hay supply and a higher NDF level, increased fermentation in the large intestine, and promoted higher growth of different bacterial groups. The low intake observed by Poczynek et al. [13] can also influence fecal bacterial diversity. Thus, the low intake associated with a slower ruminal development would allow more dietary substrate to reach the gut, as ruminal fermentation was not efficient. Perhaps diets affected fecal bacterial diversity more in the pre-weaning period. However, after weaning (week 10), both the structure and diversity were very similar to week 8, when the rumen was fully developed as shown by Poczynek et al.’s [13] data. Thus, even before our study, the influence of supplementation with hay on the fecal bacterial community was not clear.

Different from the results found in the rumen, the diversity in the fecal bacterial community was affected by the different solid diets. Although, in the rumen, the absence of effects of solid diet may be associated with the presence of monensin, since this ionophore reaches very low concentrations in the gut [46,50], the higher inclusion of NDF in the solid diet of the animals significantly affected the fecal bacterial community.

According to Dill-McFarland et al. [42], bacterial communities quickly establish themselves in the gut (feces used as a proxy) of calves as early as 2 weeks of age, as observed in previous studies [5,51]. In the present study, at week 1 of age, there was already a well-established microbiota in the animals’ gut, even with less diversity and evenness than the subsequent weeks. One hypothesis may be related to the restricted environment, with animals being housed in individual suspended cages without the presence of bedding. However, there were no studies in the literature that compared how different rearing systems can affect the GIT bacteriome in dairy calves. In humans, it is already known that the environment is one of the main factors that help to modulate the gut microbiome [52,53].

The lower general diversity observed at weeks 1 and 2 of age can be attributed to the higher occurrence of diarrhea, as mentioned by Poczynek et al. [13]. Similar results were discussed by Nakamura et al. [54], when diarrheal calves had a lower microbial density and richness and, consequently, less diversity. Changes in the GIT microbiome due to diarrhea have also been identified in young cattle [55]. Moreover, treatment with antibiotics has a great effect on the intestinal/fecal microbiota [56]. In lactating cows, Ji et al. [57] noted that the use of antibiotics affects the ruminal and fecal microbiota from the third day of use, with the possibility of a residual effect for more than 18 days. Perhaps in growing animals, this effect occurs faster, but with a shorter duration, because at week 4 of age, a higher diversity and evenness were observed compared to samples from the diarrhea period. However, Dill-McFarland et al. [42] also observed less diversity in the animals’ 2nd week of life, when compared to the 4th and 8th weeks, without attributing this to another specific factor, besides the age itself.

### 4.3. Microbial Composition of GIT

Firmicutes and Bacteroidetes are the dominant bacterial phyla in the GIT [45]. In a ruminal environment, Firmicutes are typically more abundant in a predominantly forage-based diet, while Bacteroidetes are generally more abundant in diets consisting mainly of a starter concentrate [58,59]. Regardless of the level or source of fiber, firmicutes were the predominant phylum in ruminal samples during 10 weeks of life, and probably the difference of nine percentage points between diets (22NDF to 31NDF) was not enough to modulate the bacteriome, especially in animals with the developing rumen. It is known that diet has a great influence on the composition of the ruminal microbiota [60]. However, there was a strong specificity of the bacterial community with the host, which makes the composition of the bacterial community variable among calves fed the same diet [1,2]. In our study, an individual variation was observed for animals on the same diet, and could indicate the presence of other factors that may interfere in the microbial composition of the GIT.

According to Jami et al. [2], with the increase in the age of the calves, the ruminal microbial communities tend to show a decrease in the abundance of proteobacteria and, simultaneously, an increase in Bacteroidota and firmicutes from birth to weaning. However, the results of the present study showed that the three phyla mentioned above had a constant abundance throughout the age of the calves, which suggested once again the effect of including monensin in the solid diet on the ruminal bacteriome. On the other hand, this effect was observed in the fecal bacteriome, with an increase in the abundance of the phylum Bacteroidota and a decrease in Proteobacteria until week 8.

At the genus level, it was observed that *Prevotella* was predominant in the ruminal and fecal microbiome, regardless of diet. In other studies [1,4,5,39,42], this genus was also the most abundant in GIT in the pre-weaning phase. These bacteria are versatile in taking advantage of different substrates, including the fermentation of starches and xylans to produce propionate, succinate, and acetate [61]. Other studies showed that this genus rapidly increased with a solid diet intake [41,51] and in animals fed high fiber diets [2]. In the rumen, the abundance doubled from the 2nd to the 4th week of age, probably due to the increase in the starter concentrate intake, from about 50 g to almost 300 g in the 4th week [13]. As the animals presented a total solid diet intake, starter intake, and intake of NFC and TDN without differences among treatments in the pre-weaning phase [13], this could be a reason for this genus not differing from the other treatments, both in ruminal fluid samples and in feces. According to Callaway and Russell [62], strains of *Prevotella* can have a growth capacity in the presence of monensin. This would explain the high abundance of this genus, and also its high abundance in the rumen in comparison to feces.

## 5. Conclusions

The levels and sources of NDF in the solid diet were not effective in modifying the ruminal bacteriome of young calves. It is not clear whether the developing ruminal bacteriome was resilient to dietary changes or whether monensin, even in a low concentration in the solid diet, can cause effects on colonization and the establishment of the microbial community. On the other hand, the supply of hay in the solid diet resulted in a greater α diversity in the microbiome in fecal samples from dairy calves, suggesting important effects on the intestinal microbiome. In addition, the difference according to the animal’s age can be a tool to understand the better moment for potential dietary interventions to manipulate the composition of the gut bacteriome to improve animal performance and health.

## Figures and Tables

**Table 1:** Ingredient and chemical composition of experimental diets and hay.

Item	22NDF	31NDF	Hay
Chemical composition, g/Kg			
DM	887	895	885
Ash	83	87	72
CP	185	189	118
NDF	220	310	696
ADF	119	202	401
Lignin	83	12	53
EE	28	26	13
NFC	486	401	-
TDN	848	789	540
Ingredient, g/Kg			
Ground corn	560	420	-
Soybean meal	290	270	-
Soybean hulls	110	270	-
Mineral/vitamin premix ^1^	40	40	-

^1^ Composition: Ca, 20%; P, 6.5%; In, 4%; K, 1%; Mg, 7%; S, 0.7%; F, 650 ppm; Co, 25 ppm; Cu, 800 ppm; Cr, 20 ppm; I, 40 ppm; Fe, 1400 ppm; Mn, 1500 ppm; Se, 18 ppm; Zn, 3200 ppm; Vit. A, 140,000 IU/kg; Vit. D3, 50,000 IU/kg; Vit. E, 1500 IU/kg; Vit. B1, 250 ppm, Vit. B2, 250,000 ppm; Vit. B6, 250 ppm; Vit. B12, 250 ppm; niacin, 400 ppm; pantothenic acid, 500 ppm; folic acid, 20 ppm; biotin, 10 ppm; B.H.T (butylated hydroxytoluene), 800 ppm; sodium monensin, 900 ppm.

**Table 2:** α-diversity in ruminal fluid of dairy calves fed solid diets containing different levels and sources of NDF.

Indices	Diet ^1^			SEM	*p*-Value		
	22Hay	22NDF	31NDF		D ^2^	A ^3^	DxA ^4^
Shannon	2.26	2.37	2.35	0.076	0.555	0.776	0.748
Simpson	0.80	0.82	0.82	0.017	0.658	0.731	0.850
Chao1	56.52	54.66	50.61	3.506	0.708	0.137	0.322
Pielou	0.57	0.60	0.59	0.016	0.470	0.857	0.953

^1^ 22NDF—calves fed starter concentrate with 220 g NDF/kg; 22Hay—calves fed starter concentrate with 220 g NDF/kg and grass hay; 31NDF—calves fed starter concentrate containing 310 g NDF/kg; ^2^ D—diet; ^3^ A—age; ^4^ DxI—interaction between diet and age.

**Table 3:** α-diversity in fecal content of dairy calves fed solid diets containing different levels and sources of NDF.

Indices	Diet ^1^			SEM	*p*-Value		
	22Hay	22NDF	31NDF		D ^2^	A ^3^	DxA ^4^
Shannon	2.80 ^a^	2.58 ^b^	2.71 ^ab^	0.105	0.048	<0.001	0.309
Simpson	0.88 ^a^	0.84 ^b^	0.85 ^ab^	0.014	0.072	<0.001	0.481
Chao1	69.64	73.48	75.54	7.166	0.668	<0.001	0.278
Pielou	0.67 ^a^	0.61 ^b^	0.64 ^ab^	0.016	0.007	<0.001	0.301

^ab^ Means within a row with different superscripts are significantly different (*p* ≤ 0.05); ^1^ 22NDF—calves fed starter concentrate with 220 g NDF/kg; 22Hay—calves fed starter concentrate with 220 g NDF/kg and grass hay; 31NDF—calves fed starter concentrate containing 310 g NDF/kg; ^2^ D—diet;^3^ A—age; ^4^ DxI—interaction between diet and age.

## Data Availability

All raw DNA sequence reads were deposited in NCBI’s Sequence Read Archive under BioProject PRJNA639165, submission SUB7605036.

## Supplementary Materials

The following are available online at https://www.mdpi.com/article/10.3390/ani11092705/s1, Supplementary Table S1: Sequencing information. Supplementary Table S2: Taxon identification and counting of fecal samples collected of calves fed different solid diet levels and sources of NDF. Supplementary Table S3: Taxon identification and counting of ruminal fluid samples collected of calves fed different solid diet levels and sources of NDF. Supplementary Table S4: Abundance of phyla over the weeks of ruminal fluid samples collected of calves fed different solid diet levels and sources of NDF. Supplementary Table S5: Abundance of genera over the weeks of ruminal fluid samples collected of calves fed different solid diet levels and sources of NDF. Supplementary Table S6: Differential abundance of phyla and genus over the weeks of ruminal fluid samples collected of calves fed different solid diet levels and sources of NDF. Supplementary Table S7: Abundance of phyla over the weeks of feces samples collected of calves fed different solid diet levels and sources of NDF. Supplementary Table S8: Abundance of genera over the weeks of feces samples collected of calves fed different solid diet levels and sources of NDF. Supplementary Table S9: Differential abundance of phyla and genus over the weeks of feces collected of calves fed different solid diet levels and sources of NDF.

## References

[1] Li R.W., Connor E.E., Li C., Vi R.L.B., Sparks M.E. (2012). Characterization of the rumen microbiota of pre-ruminant calves using metagenomic tools. Environ. Microbiol..

[2] Jami E., Israel A., Kotser A., Mizrahi I. (2013). Exploring the bovine rumen bacterial community from birth to adulthood. ISME J..

[3] Jiao J., Huang J., Zhou C., Tan Z. (2015). Taxonomic identification of ruminal epithelial bacterial diversity during rumen development in goats. Appl. Environ. Microbiol..

[4] Dias J., Marcondes M.I., Noronha M.F., Resende R.T., Machado F.S., Mantovani H.C., Dill-McFarland K.A., Suen G. (2017). Effect of pre-weaning diet on the ruminal archaeal, bacterial, and fungal communities of dairy calves. Front. Microbiol..

[5] Uyeno Y., Sekiguchi Y., Tajima K., Takenaka A., Kurihara M., Kamagata Y. (2010). An rRNA-based analysis for evaluating the effect of heat stress on the rumen microbial composition of Holstein heifers. Anaerobe.

[6] Oikonomou G., Teixeira A.G.V., Foditsch C., Bicalho M.L., Machado V.S., Bicalho R.C. (2013). Fecal microbial diversity in pre-weaned dairy calves as described by pyrosequencing of metagenomic 16S rDNA. Associations of faecalibacterium species with health and growth. PLoS ONE.

[7] Myer P.R., Wells J.E., Smith T.P.L., Kuehn L.A., Freetly H.C. (2016). Microbial community profiles of the jejunum from steers differing in feed efficiency1,2,3. J. Anim. Sci..

[8] Dias J., Marcondes M.I., de Souza S.M., da Mata e Silva B.C., Noronha M.F., Resende R.T., Machado F.S., Mantovani H.C., Dill-McFarland K.A., Suen G. (2018). Bacterial community dynamics across the gastrointestinal tracts of dairy calves during preweaning development. Appl. Environ. Microbiol..

[9] Biesheuvel M.H., Bijker P.G.H., Urlings H.A.P. (1991). Some aspects of the gastrointestinal microflora of veal calves fed different rations: A pilot study. Vet. Q..

[10] Carberry C.A., Waters S.M., Kenny D.A., Creevey C.J. (2014). Rumen methanogenic genotypes differ in abundance according to host residual feed intake phenotype and diet type. Appl. Environ. Microbiol..

[11] Khan M.A., Bach A., Weary D.M., von Keyserlingk M.A.G. (2016). Invited review: Transitioning from milk to solid feed in dairy heifers. J. Dairy Sci..

[12] Diao Q., Zhang R., Fu T. (2019). Review of Strategies to Promote Rumen Development in Calves. Animals.

[13] Poczynek M., Toledo A.F., Silva A.P., Silva M.D., Oliveira G.B., Coelho M.G., Virginio G.F., Polizel D., Costa J.H.C., Bittar C.M.M. (2020). Partial corn replacement by soybean hull, or hay supplementation: Effects of increased NDF in diet on performance, metabolism and behavior of pre-weaned calves. Livest. Sci..

[14] Davis C.L., Drackley J.K., Edn (1998). The Development, Nutrition, and Management of the Young Calf.

[15] Daneshvar D., Khorvash M., Ghasemi E., Mahdavi A.H., Moshiri B., Mirzaei M., Pezeshki A., Ghaffari M.H. (2015). The effect of restricted milk feeding through conventional or step-down methods with or without forage provision in starter feed on performance of Holstein bull calves1. J. Anim. Sci..

[16] Pounden W.D., Hibbs J.W. (1948). The Influence of the ratio of grain to hay in the ration of dairy calves on certain rumen microorganisms. J. Dairy Sci..

[17] Hibbs J.W., Conrad H.R., Pounden W.D., Frank N. (1956). A high roughage system for raising calves based on early development of rumen function. VI. Influence of hay to grain ratio on calf performance, rumen development, and certain blood changes. J. Dairy Sci..

[18] Bryant M.P., Small N., Bouma C., Robinson I. (1958). Studies on the composition of the ruminal flora and fauna of young calves. J. Dairy Sci..

[19] Zambom M.A., dos Santos G.T., Modesto E.C., Alcalde C.R., Gonçalves G.D., da Silva D.C., da Silva K.T., Faustino J.O. (2001). Valor nutricional da casca do grão de soja, farelo de soja, milho moído e farelo de trigo para bovinos. Acta Sci..

[20] Oltramari C.E., Nápoles G.G.O., De Paula M.R., Silva J.T., Gallo M.P.C., Soares M.C., Bittar C.M.M. (2016). Performance and metabolism of dairy calves fed starter feed containing citrus pulp as a replacement for corn. Anim. Prod. Sci..

[21] Cunningham K.D., Cecava M.J., Johnson T.R. (1993). Nutrient digestion, nitrogen, and amino acid flows in lactating cows fed soybean hulls in place of forage or concentrate. J. Dairy Sci..

[22] Godden S. (2008). Colostrum management for dairy calves. Vet. Clin. N. Am. Food Anim. Pract..

[23] Elsohaby I., McClure J.T., Waite L.A., Cameron M., Heider L.C., Keefe G.P. (2019). Using serum and plasma samples to assess failure of transfer of passive immunity in dairy calves. J. Dairy Sci..

[24] NRC (2001). Nutrient Requirements of Dairy Cattle.

[25] Yu Z., Morrison M. (2004). Improved extraction of PCR-quality community DNA from digesta and fecal samples. Biotechniques.

[26] Callahan B.J., McMurdie P.J., Rosen M.J., Han A.W., Johnson A.J.A., Holmes S.P. (2016). DADA2: High-resolution sample inference from Illumina amplicon data. Nat. Methods.

[27] Gentleman R.C., Carey V.J., Bates D.M., Bolstad B., Dettling M., Dudoit S., Ellis B., Gautier L., Ge Y., Gentry J. (2004). Bioconductor: Open software development for computational biology and bioinformatics. Genome Biol..

[28] Huber W., Carey V.J., Gentleman R., Anders S., Carlson M., Carvalho B.S., Bravo H.C., Davis S., Gatto L., Girke T. (2015). Orchestrating high-throughput genomic analysis with Bioconductor. Nat. Methods.

[29] Callahan B.J., Sankaran K., Fukuyama J.A., McMurdie P.J., Holmes S.P. (2016). Bioconductor workflow for microbiome data analysis: From raw reads to community analyses. F1000Research.

[30] Wang Q., Garrity G.M., Tiedje J.M., Cole J.R. (2007). Naïve bayesian classifier for rapid assignment of rRNA sequences into the new bacterial taxonomy. Appl. Environ. Microbiol..

[31] Glöckner F.O., Yilmaz P., Quast C., Gerken J., Beccati A., Ciuprina A., Bruns G., Yarza P., Peplies J., Westram R. (2017). 25 years of serving the community with ribosomal RNA gene reference databases and tools. J. Biotechnol..

[32] McMurdie P.J., Holmes S. (2013). phyloseq: An R package for reproducible interactive analysis and graphics of microbiome census data. PLoS ONE.

[33] Robinson M.D., McCarthy D.J., Smyth G.K. (2010). edgeR: A Bioconductor package for differential expression analysis of digital gene expression data. Bioinformatics.

[34] Robinson M.D., Oshlack A. (2010). A scaling normalization method for differential expression analysis of RNA-seq data. Genome Biol..

[35] Law C.W., Chen Y., Shi W., Smyth G.K. (2014). Voom: Precision weights unlock linear model analysis tools for RNA-seq read counts. Genome Biol..

[36] Ritchie M.E., Phipson B., Wu D., Hu Y., Law C.W., Shi W., Smyth G.K. (2015). Limma powers differential expression analyses for RNA-sequencing and microarray studies. Nucleic Acids Res..

[37] Smyth G.K. (2004). Linear models and empirical bayes methods for assessing differential expression in microarray experiments. Stat. Appl. Genet. Mol. Biol..

[38] Malmuthuge N., Guan L.L. (2017). Understanding the gut microbiome of dairy calves: Opportunities to improve early-life gut health. J. Dairy Sci..

[39] Biscarini F., Palazzo F., Castellani F., Masetti G., Grotta L., Cichelli A., Martino G. (2018). Rumen microbiome in dairy calves fed copper and grape-pomace dietary supplementations: Composition and predicted functional profile. PLoS ONE.

[40] Kim Y.-H., Nagata R., Ohtani N., Ichijo T., Ikuta K., Sato S. (2016). Effects of dietary forage and calf starter diet on ruminal pH and bacteria in Holstein calves during weaning transition. Front. Microbiol..

[41] Rey M., Enjalbert F., Combes S., Cauquil L., Bouchez O., Monteils V. (2014). Establishment of ruminal bacterial community in dairy calves from birth to weaning is sequential. J. Appl. Microbiol..

[42] Dill-McFarland K.A., Breaker J.D., Suen G. (2017). Microbial succession in the gastrointestinal tract of dairy cows from 2 weeks to first lactation. Sci. Rep..

[43] Schelling G.T. (1984). Monensin mode of action in the rumen. J. Anim. Sci..

[44] Bangoura B., Bardsley K.D. (2020). Ruminant coccidiosis. Vet. Clin. N. Am. Food Anim. Pract..

[45] Clemmons B.A., Voy B.H., Myer P.R. (2019). Altering the gut microbiome of cattle: Considerations of host-microbiome interactions for persistent microbiome manipulation. Microb. Ecol..

[46] Thomas M., Webb M., Ghimire S., Blair A., Olson K., Fenske G.J., Fonder A.T., Christopher-Hennings J., Brake D., Scaria J. (2017). Metagenomic characterization of the effect of feed additives on the gut microbiome and antibiotic resistome of feedlot cattle. Sci. Rep..

[47] Poe S.E., Ely D.G., Mitchell G.E., Deweese W.P., Glimp H.A. (1971). Rumen development in lambs: I. Microbial digestion of starch and cellulose. J. Anim. Sci..

[48] Gagen E.J., Mosoni P., Denman S.E., Jassim R.A., McSweeney C.S., Forano E. (2012). Methanogen colonisation does not significantly alter acetogen diversity in lambs isolated 17 h after birth and raised aseptically. Microb. Ecol..

[49] Guzman C.E., Bereza-Malcolm L.T., Groef B.D., Franks A.E. (2015). Presence of selected methanogens, fibrolytic bacteria, and proteobacteria in the gastrointestinal tract of neonatal dairy calves from birth to 72 hours. PLoS ONE.

[50] Herberg R., Manthey J., Richardson L., Cooley C., Donoho A. (1978). Excretion and tissue distribution of [14C]monensin in cattle. J. Agric. Food Chem..

[51] Malmuthuge N., Griebel P.J., Guan L.L. (2014). Taxonomic identification of commensal bacteria associated with the mucosa and digesta throughout the gastrointestinal tracts of preweaned calves. Appl. Environ. Microbiol..

[52] Korpela K., Salonen A., Virta L.J., Kekkonen R.A., Forslund K., Bork P., de Vos W.M. (2016). Intestinal microbiome is related to lifetime antibiotic use in Finnish pre-school children. Nat. Commun..

[53] Dong T.S., Gupta A. (2019). Influence of early life, diet, and the environment on the microbiome. Clin. Gastroenterol. Hepatol..

[54] Nakamura S.-I., Kim Y.H., Takashima K., Kimura A., Nagai K., Ichijo T., Sato S. (2017). Composition of the microbiota in forestomach fluids and feces of Japanese Black calves with white scours. J. Anim. Sci..

[55] Zeineldin M., Aldridge B., Lowe J. (2018). Dysbiosis of the fecal microbiota in feedlot cattle with hemorrhagic diarrhea. Microb. Pathog..

[56] Oultram J., Phipps E., Teixeira A.G.V., Foditsch C., Bicalho M.L., Machado V.S., Bicalho R.C., Oikonomou G. (2015). Effects of antibiotics (oxytetracycline, florfenicol or tulathromycin) on neonatal calves’ faecal microbial diversity. Vet. Rec..

[57] Ji S., Jiang T., Yan H., Guo C., Liu J., Su H., Alugongo G.M., Shi H., Wang Y., Cao Z. (2018). Ecological restoration of antibiotic-disturbed gastrointestinal microbiota in foregut and hindgut of cows. Front. Cell. Infect. Microbiol..

[58] Fernando S.C., Purvis H.T., Najar F.Z., Sukharnikov L.O., Krehbiel C.R., Nagaraja T.G., Roe B.A., DeSilva U. (2010). Rumen microbial population dynamics during adaptation to a high-grain diet. Appl. Environ. Microbiol..

[59] McCann J.C., Luan S., Cardoso F.C., Derakhshani H., Khafipour E., Loor J.J. (2016). Induction of subacute ruminal acidosis affects the ruminal microbiome and epithelium. Front. Microbiol..

[60] Thoetkiattikul H., Mhuantong W., Laothanachareon T., Tangphatsornruang S., Pattarajinda V., Eurwilaichitr L., Champreda V. (2013). Comparative analysis of microbial profiles in cow rumen fed with different dietary fiber by tagged 16S rRNA gene pyrosequencing. Curr. Microbiol..

[61] Flint H.J., Bayer E.A., Rincon M.T., Lamed R., White B.A. (2008). Polysaccharide utilization by gut bacteria: Potential for new insights from genomic analysis. Nat. Rev. Microbiol..

[62] Callaway T.R., Russell J.B. (2000). Variations in the ability of ruminal gram-negative prevotella species to resist monensin. Curr. Microbiol..

